# Blood-Derived microRNAs for Pancreatic Cancer Diagnosis: A Narrative Review and Meta-Analysis

**DOI:** 10.3389/fphys.2018.00685

**Published:** 2018-06-05

**Authors:** Xiaodong Li, Pujun Gao, Yang Wang, Xiaocong Wang

**Affiliations:** ^1^Suzhou Institute of Biomedical Engineering and Technology, Chinese Academy of Sciences, Suzhou, China; ^2^University of Chinese Academy of Sciences, Beijing, China; ^3^Department of Echocardiography, The First Hospital of Jilin University, Changchun, China; ^4^Department of Hepatology, The First Hospital of Jilin University, Changchun, China; ^5^Department of Orthopedics, China-Japan Friendship Hospital, Changchun, China

**Keywords:** microRNAs, pancreatic cancer, diagnosis, differential, blood, meta-analysis

## Abstract

microRNAs (miRNAs) have been reported to be aberrantly expressed in patients with pancreatic cancer. In present review we explored the biological roles of miRNAs in pancreatic cancer and their clinical value in diagnosis. In the systematic review, the potential value of miRNAs as biomarkers was investigated by reviewing the altered miRNA profiles reported in pancreatic cancer patients in 356 included studies. In the subsequent meta-analysis, we included 17 studies in early diagnosis of pancreatic cancer with a panel of altered miRNAs. The following results were obtained: pooled sensitivity of 0.88 (95% confidence interval [CI] 0.83–0.92), pooled specificity of 0.83 (95%CI 0.77–0.88), diagnostic odds ratio of 27 (95%CI 14–53), and area under the receiver operating characteristic curve of 0.90 (95%CI 0.88–0.93). To further explore the value of a single miRNA, the diagnostic value of miR-21 in PA was also demonstrated by the pooled sensitivity (0.90, 95% CI: 0.82–0.94), specificity (0.72, 95% CI: 0.57-0.83) as well as AUC (0.91 (95%CI 0.88–0.93). In conclusion, our findings suggest that aberrant miRNA expression in blood play an essential role in pancreatic cancer, and meta-analysis revealed blood-derived miRNAs as probable biomarkers for early diagnosis of pancreatic cancer.

## Introduction

Pancreatic cancer is one of the most malignant gastrointestinal (GI) tumors and thus has a poor prognosis, with the mortality of this disease ranking fourth among cancer-related deaths (Real, 2003). Due to the lack of early clinical manifestations and occult onset, most patients are already in advanced stages when diagnosed with pancreatic cancer, showing aggressive growth and spreading of the tumor, and therefore cannot be surgically cured (Parikh and Lillemoe, 2015). In addition, pancreatic cancer tumors are typically resistant to radiotherapy and chemotherapy, resulting a lower 5-year survival rate and high mortality (Govindan and Goldenberg, 2015). Currently, the diagnosis of pancreatic cancer depends mainly on nonspecific tumor biomarkers. However, due to their non-specificity, patients cannot be promptly diagnosed and receive proper treatment, thus greatly reducing patients' survival rate (Schmidt, 2012). Therefore, the identification of new types of pancreatic cancer biomarkers is essential for clinicians to improve the survival of pancreatic cancer patients.

microRNAs (miRNAs) are a class of single-stranded non-coding RNAs that are involved in the regulation of physical and pathological processes such as cell proliferation, protein metabolism, and tumor development. They are highly conserved and tissue-specific. Through binding to the 3′-untranslational region (UTR) of target mRNAs, miRNAs degrade or inhibit the translation of mRNA, a process known as post-transcriptional silencing, and participate in the regulation of target gene functions (Liu, 2014). In recent years, miRNAs have been reported to be associated with tumorigenesis of blood cancers and solid tumors (Garzon et al., 2015). In pancreatic cancer, the up-regulation or down-regulation of miRNAs is closely related to the biological characteristics of the tumor as well as tumor metastasis and prognosis (Ren et al., 2012).

We conducted the present review of relevant qualitative and quantitative studies to identify altered miRNA regulation with previous findings on patients with pancreatic cancer. This was followed by a meta-analysis to evaluate the early diagnostic value with altered miRNA profiles for pancreatic cancer.

## Materials and methods

### Literature search strategy

A comprehensive literature search was carried out for studies reporting the association between circulating miRNAs and pancreatic cancer in patients through the following databases: PubMed, Embase, the Cochrane Library, and Web of Science. In addition, we searched the Chinese databases including Chinese National Knowledge Infrastructure (CNKI) database, Technology of Chongqing VIP database, and Wan Fang Data. We used search terms with the key words: “pancreatic cancer” “pancreatic ductal adenocarcinoma,” “pancreatic neoplasm” “pancreatic tumor” “pancreatic carcinoma,” “microRNA,” “miRNA,” “diagnosis,” “specificity,” “sensitivity,” and “ROC curve” up to February 1, 2018. In addition, we searched conference abstracts, summaries and references of reviews to find any potentially eligible articles.

### Inclusion criteria

In the narrative review, we included quantitative and qualitative studies investigating the correlation between miRNA expression and pancreatic cancer, including reviews, letters, reports, editorials, research articles, or conference abstracts.

In the meta-analysis, included studies had to meet the following criteria: (1) randomized case-control study on patients with pancreatic cancer, (2) research on the association between circulating miRNAs and pancreatic cancer, and (3) provided sufficient data that could be calculated or extracted from the article.

The exclusion criteria were: animal model research, reviews, commentaries, editorials, or duplicate publications, unrelated to pancreatic cancer and miRNAs, provided limited data, or overlap of patient selection with another study.

### Data extraction and quality assessment

The following data were extracted by two of the authors independently from the full text of eligible articles: first author's name; year and country of publication; sample size, sex, and miRNA expression signatures; and specificity and sensitity values of tested miRNAs. The quality of the included studies was systematically evaluated according to QUADAS-2 guidelines for the qualitative assessment of diagnostic accuracy studies (Whiting et al., 2006; Kojimahara et al., 2014).

### Statistical analysis of meta-analysis

Stata statistical software and Meta-DiSc 1.4 were used for data analyses. The true positive (TP), false positive (FP), true negative (TN), and false negative (FN) rates were calculated for the obtained data. Heterogeneity across studies was assessed based on the χ^2^-based Cochran's Q test and Higgins' *I*^2^ statistics. The bivariate binomial mixed model was used to calculate the pooled sensitivity, specificity, diagnostic odds ratio (DOR), positive likelihood ratio (PLR), and negative likelihood ratio (NLR) with 95% confidence intervals (CIs). A summary receiver operating characteristic curve (SROC) was constructed based on the pooled sensitivity and specificity. The area under the SROC curve (AUC) provided the accuracy of a quantitative diagnostic test with miRNAs for pancreatic cancer. In addition, to explore the potential heterogeneity among included studies, subgroup analyses and meta-regression were performed. Deek's funnel plots were adopted to examine publication bias across studies. All statistical tests were two-sided with significance level set as p < 0.05.

## Results

### Narrative review: relationship between miRNA expression and pancreatic cancer

#### Altered expression of miRNAs in pancreatic cancer

miRNAs regulate target gene expression, and their expression in cancer tissues and normal tissues can differ significantly. In oncology, miRNAs are now regarded as oncogenes or tumor suppressor genes. It is generally believed that miRNAs that are up-regulated in tumor tissue are oncogenes that promote tumor development by regulating cell differentiation or apoptosis via inhibiting tumor suppressor genes. Bloomston et al. using miRNA chips and real-time PCR identified seven miRNAs (miR-21, miR-221, miR-222, miR-181a, miR-181b, miR-181d, and miR-155) that were up-regulated in pancreatic cancer tissues (Bloomston et al., 2007). Additionally, Volinia et al. aanalyzed pancreatic tumors in a large number of patients through RNA-seq technology and found miRNAs that were expressed at higher levels in tumors (miR-17-5p, miR-20a, miR-21, miR-92, miR-106a, and miR-155) (Volinia et al., 2006).

Some miRNAs are also downregulated in pancreatic cancers where they regulate tumor suppressor genes; for example, miR-34 was shown to be down-regulated in pancreatic cancer cell lines. Ji et al. found that miR-34a expression in at least two pancreatic cancer cell lines was reduced by at least 2 fold, and in 11 pancreatic cancer cell lines, miR-34a expression was even reduced by more than 10 fold (Ji et al., 2009). In addition, Lodygin et al. found that miR-34 expression in the MiaPaCa-2 and Bxpc-3 pancreatic cancer cell lines was extremely reduced (Lodygin et al., 2008). Saito et al. also found that miR-127 is down-regulated in human primary tumors compared to levels in normal cells, suggesting that miR-127 may be a potential tumor suppressor (Saito et al., 2006). Moreover, by comparing the expression levels of 241 miRNAs in 60 types of tumor cells and normal tissues from the National Cancer Institute (NCI), Gaur et al. found that most miRNAs were down-regulated in tumor cells, suggesting that most of the miRNAs may be tumor suppressors (Gaur et al., 2007). miRNA profiles in identifying pancreatic cancer and other pancreatic diseases. Some studies suggest that miR-204 is mainly expressed in insulinoma-regulating insulin secretion. Ki-67 is a marker of cell proliferation and has been linked to prognosis in many oncology studies. Up-regulation of miR-21 is associated with higher Ki-67 expression and hepatic metastasis, suggesting that miRNA changes are associated with pancreatic endocrine tumors, adenoma transformation, and malignant tumor progression. Roldo et al. screened miRNA profiles in 12 non-pancreatic tumor tissues and primary pancreatic tumors (insulinomas, non-functioning pancreatic endocrine tumors, and adenocarcinomas) and found miRNA expression patterns that differed from those in normal pancreatic tissue. Ren et al. examined the expression of 95 miRNAs involved in oncogenesis in pancreatic cancer tissues and compared miRNA signatures in normal human pancreatic ductal epithelial (HPDE) cells and normal pancreatic tissues. They found that 8 miRNAs (miR-196a, miR-190, miR-186, miR-221, miR-222, miR-200b, miR-15b, and miR-95) were abundant in pancreatic cancer tissues (Ren et al., 2012). These authors concluded that pancreatic cancer tissue or cell lines have unique miRNA expression profiles. Bloomston et al. used miRNA microarrays to investigate the expression of 326 miRNAs in 65 cases of pancreatic cancer, 42 cases of chronic pancreatitis, and normal pancreas tissue, and the results showed that compared with their expression in normal pancreatic tissue, 21 miRNAs were up-regulated and four miRNAs were down-regulated in pancreatic cancers (Bloomston et al., 2007). Fifteen miRNAs were up-regulated and eight miRNAs were down-regulated in pancreatic cancer compared with chronic pancreatitis. The expression profiles of these miRNAs were used to distinguish chronic pancreatitis and pancreatic cancer with accuracy of 93%. Subsequent cluster analysis found that miRNA expression patterns were very similar between chronic pancreatitis and normal pancreas, whereas miRNA expression was significantly different between chronic pancreatitis or normal pancreas. Therefore, miRNA expression of pancreatic cancer could be used for differential diagnosis of pancreatic cancer from benign diseases.

### miRNA profiles in identifying pancreatic cancer and other tumors

miRNA expression is strongly tissue-specific. Volinia et al. used miRNA microarray technology to examine 363 tissue samples of six types of tumors from pancreas, lung, breast, stomach, prostate, and colon, and the results revealed that the expression profile of a cluster of 137 miRNAs can be a good indicator for differentiating the source of tumor tissue (Volinia et al., 2006). A 4-year study found that different tumors have specific miRNA expression patterns, including chronic lymphocytic leukemia, gastric cancer, lung cancer, breast cancer, prostate cancer, pancreatic cancer, liver cancer, and thyroid cancer. In pancreatic tissues, up-regulation of miR-216 and miR-217 expression as well as deletion of miR-133a can be regarded as features of pancreatic tissues to determine the pancreatic origin of tissues or cells (Gironella et al., 2007).

#### Meta-analysis: diagnostic value of miRNA profile for pancreatic cancer diagnosis

**Figure 1:**
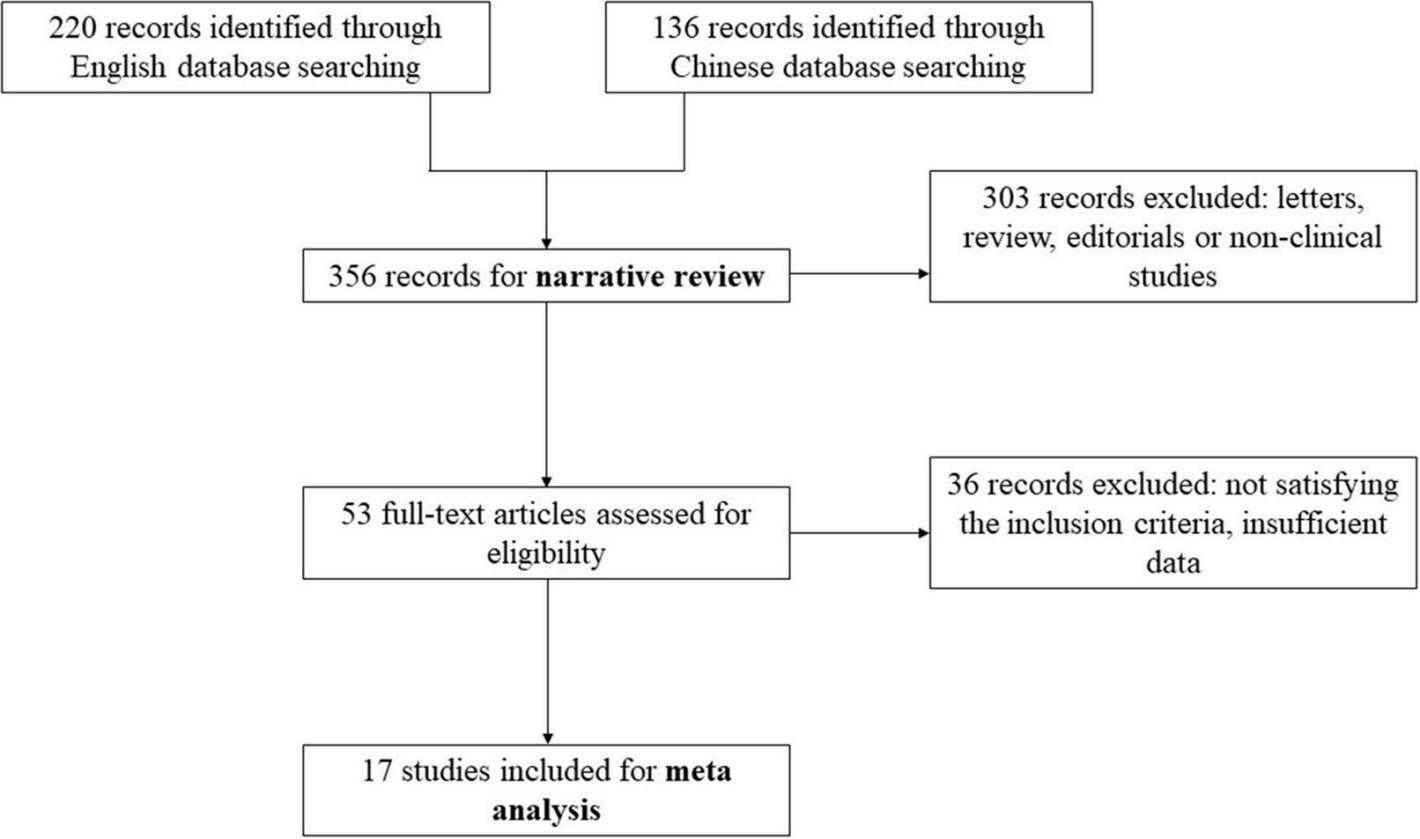
Process of study selection.

We identified 17 publications examining the clinical value of miRNA expression for the diagnosis of pancreatic cancer. The details of the selection process are presented in Figure 1. A total of 939 cases of pancreatic cancer and 977 control cases were included for the meta-analysis across 17 studies, and the study characteristics along with the QUADAS scores of the included studies are presented in Table 1. All patients with pancreatic cancer were diagnosed according to clinically recognized diagnostic criteria. Analysis of the quality of each study revealed QUADAS scores ranging from 4 to 9.

**Table 1:** Summary of included studies.

**Study (Year)**	**Country**	**No. of patients**	**No. of controls**	**Specimen**	**TP**	**FP**	**FN**	**TN**	**QUADAS**	**miRNAs**
Abue et al., 2015	Japan	32	42	Plasma	25	7	19	23	8	miR-483-3p,−21
Wang et al., 2009	USA	28	19	Plasma	18	10	2	17	6	miR-21,−210,−155,−196a
Morimura et al., 2011	Japan	36	30	Plasma	34	2	1	29	6	miR-18a
Liu et al., 2011	China	62	97	Plasma	39	23	15	82	4	miR-155
Liu et al., 2012	China	138	175	Serum	89	49	37	138	5	miR-16,−196a
Pan et al., 2012	China	24	24	Plasma	22	2	2	22	5	miR-451,−409-3p
Pan, 2014	China	30	26	Plasma	21	9	4	22	4	miR-210,−25
Shi, 2014	China	60	30	Plasma	46	14	9	21	4	miR-155,−196a
Komatsu et al., 2015	Japan	71	67	Plasma	44	27	4	63	8	miR-223
Alemar et al., 2016	Brazil	24	10	Serum	20	4	2	8	9	miR-21,−34a
Que et al., 2013	China	22	27	Serum	21	1	5	22	7	miR-21
Que et al., 2013	China	22	27	Serum	16	6	2	25	7	miR-17-5p
Cote et al., 2014	USA	77	138	Plasma	75	2	15	0	138	miR-212,−155,−106b,−30c,−10b
Schultz et al., 2014	USA	180	199	Whole blood cell	153	27	11	188	8	miR-150,−636,−145,−233
Schultz et al., 2014	USA	180	199	Whole blood cell	153	27	4	195	8	miR-26b,−34a,−122,−126,-145, 150,-223,-505,-636,-885-5p
Miyamae et al., 2015	USA	94	68	Plasma	93	1	4	64	9	miR-744
Li et al., 2013	USA	41	19	Serum	37	4	5	14	4	miR-1290
Wang et al., 2015	China	43	21	Plasma	38	5	3	18	6	miR-21
Hu et al., 2015	China	60	30	Plasma	50	10	2	28	4	miR-155,−21,−29a,−210

**Figure 2:**
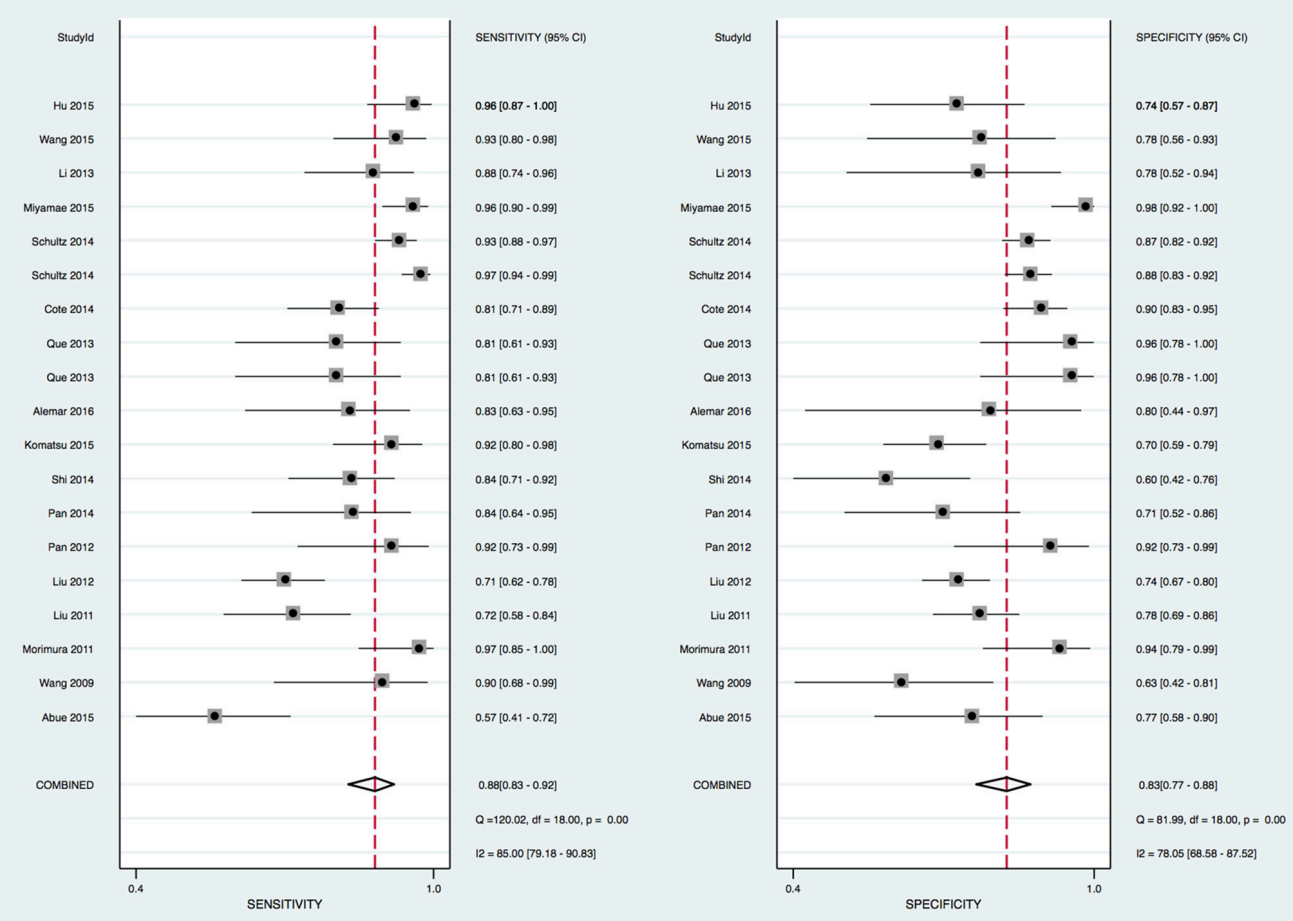
Forrest plot of estimates of sensitivity and specificity.

The heterogeneity I^2^ values for inter-study variability were 85% (sensitivity) and 78.05% (specificity). The Spearman correlation coefficient of 0.49 (*p* = 0.061) also suggested there was no heterogeneity due to the threshold effect (Figure 2).

**Figure 3:**
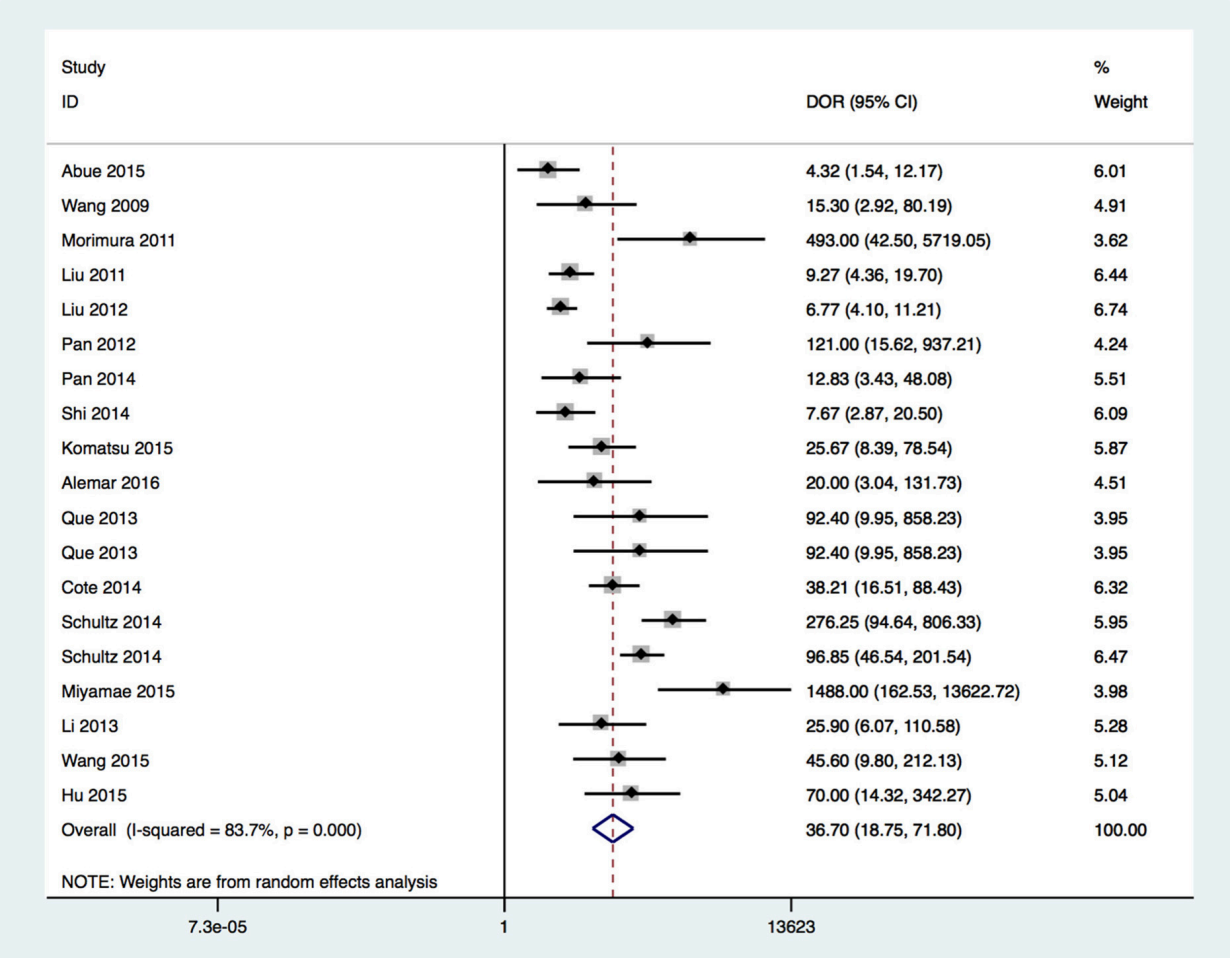
Forrest plot of estimates of DOR.

**Figure 4:**
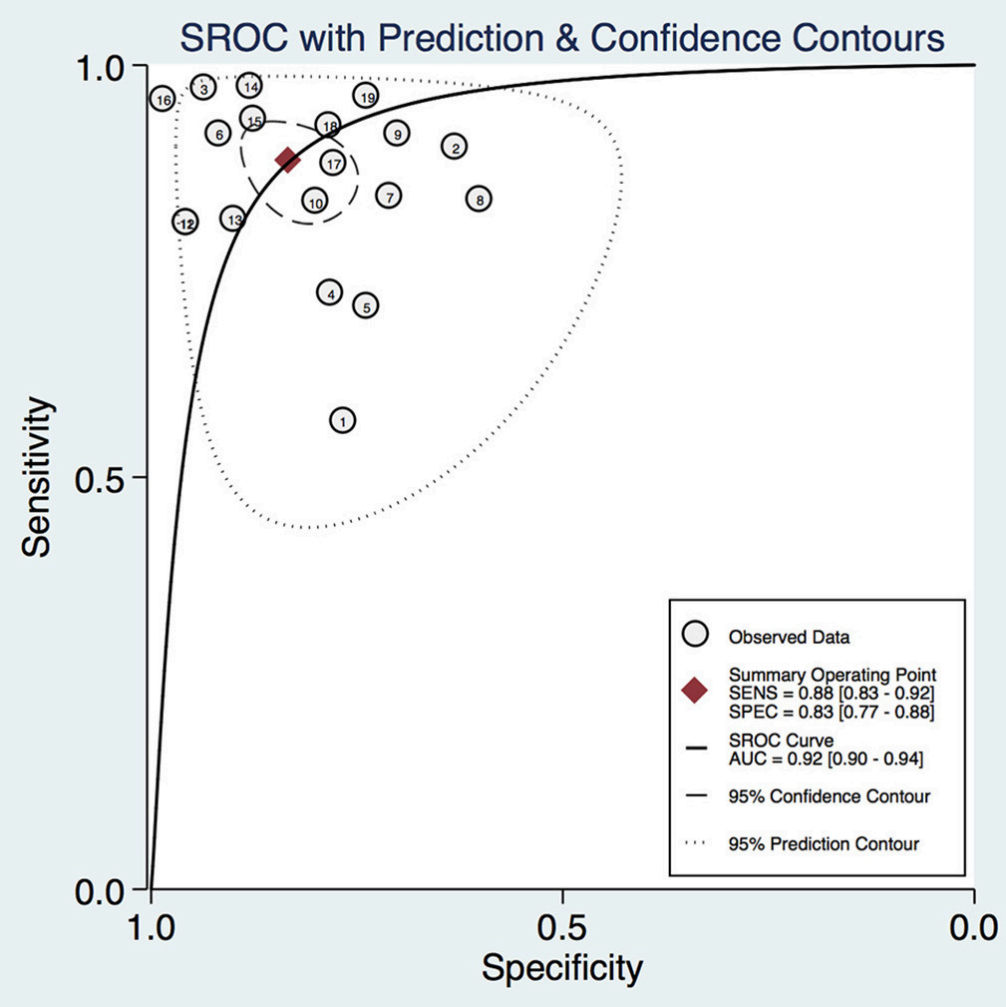
Summary receiver operating characteristic (SROC) curve.

**Figure 5:**
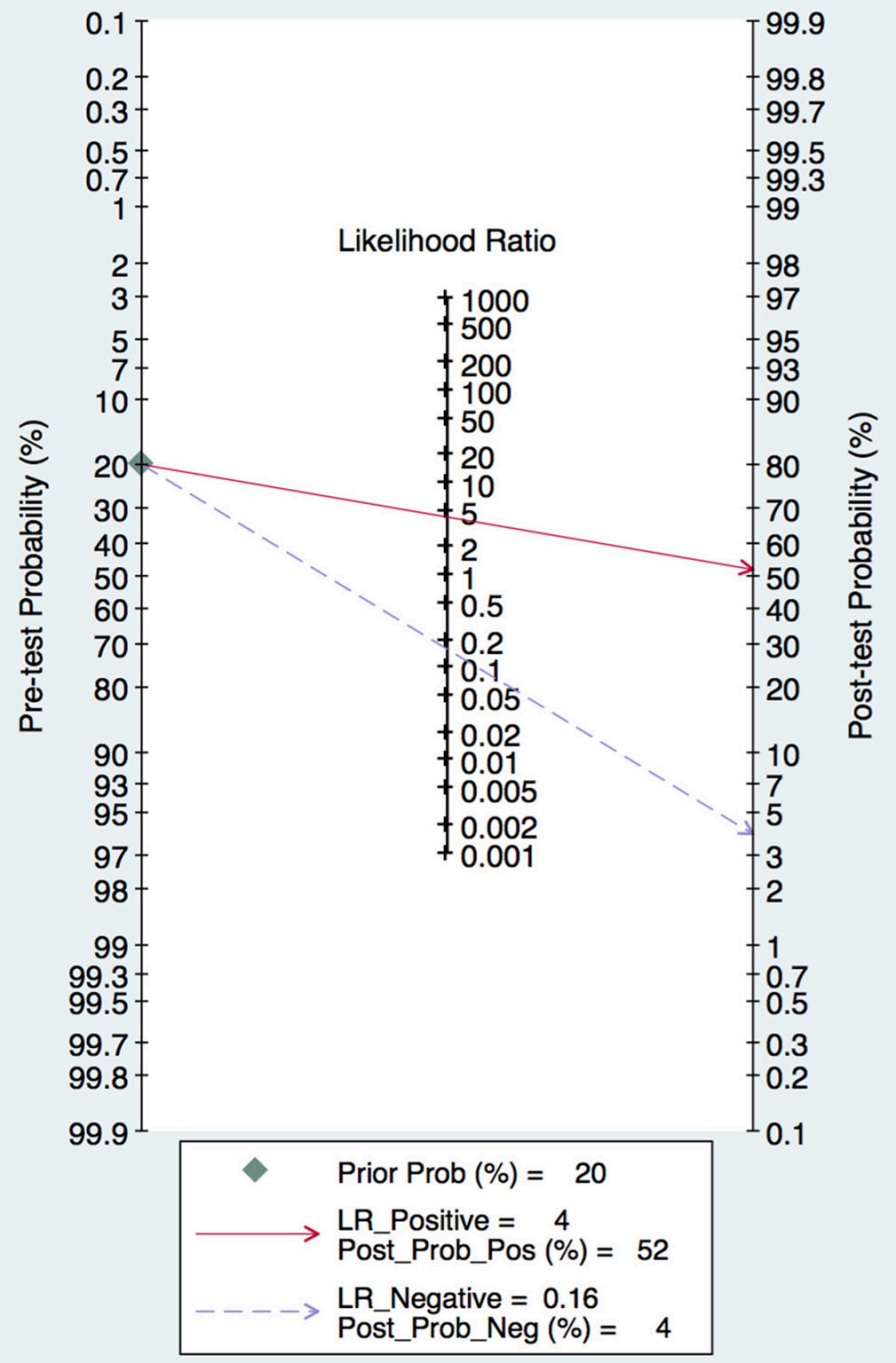
Fagan's Nomogram for assessment of post-test probabilities.

In this meta-analysis, the summary assessment of miRNAs in the diagnosis of pancreatic cancer showed that the pooled sensitivity was 0.88 (95%CI 0.83–0.92) and the pooled specificity was 0.83 (95%CI 0.77–0.88). The DOR was 36.7 (95%CI 18.75–71.80; Figure 3). The area under the SROC was 0.92 (95%CI 0.90–0.94; Figure 4). The PLR was 4.3 (95%CI 3.1–6.0), and the NLR was 0.16 (95%CI 0.11–0.24), as shown in the Fagan's nomogram in Figure 5.

**Figure 6:**
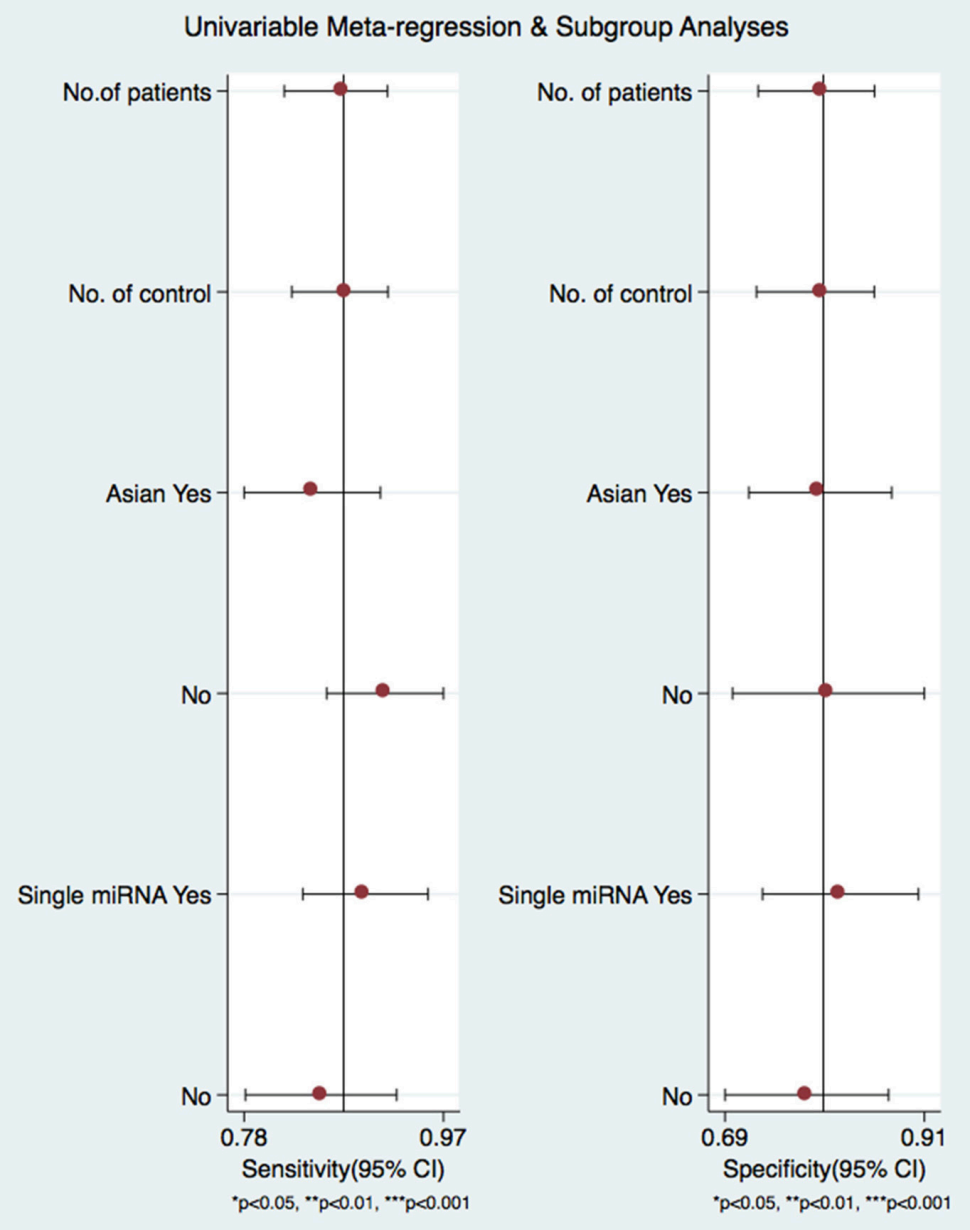
Forrest plots from multivariable meta-regression and subgroup analyses for sensitivity and specificity.

A subgroup analysis of heterogeneity sources revealed that there were no significant difference in sample size, ethnicity, or number of miRNA detected. The use of circulating miRNAs for diagnosing pancreatic cancer had a sensitivity and specificity of 0.80 and 0.89, respectively (Figure 6).

**Figure 7:**
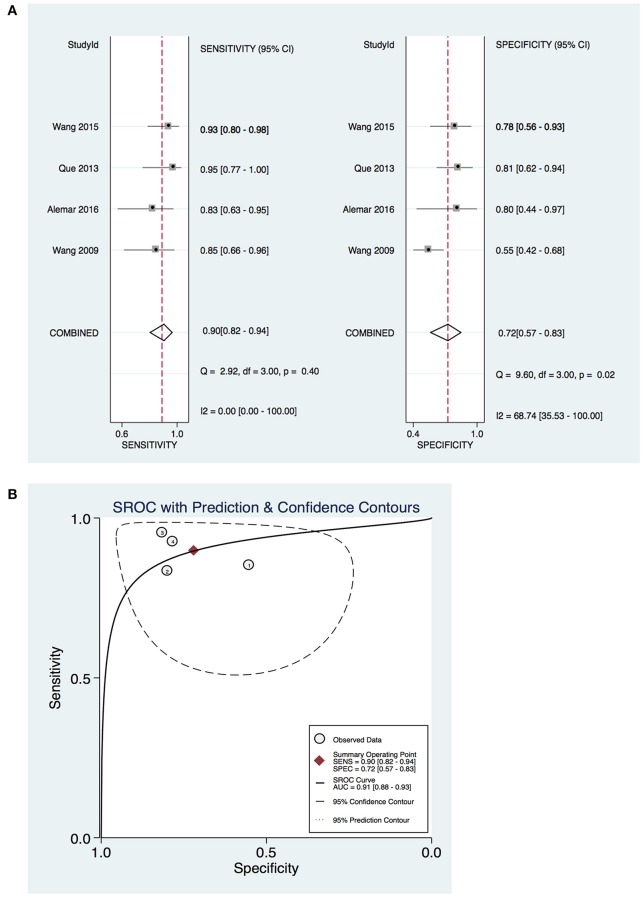
Diagnostic value of miR-21 on pancreatic cancer. **(A)** Forrest plot of estimates of sensitivity and specificity **(B)** Summary receiver operating characteristic (SROC) curve.

To further explore the diagnostic value of a single miRNA in pancreatic cancer diagnosis, we found that there were four studies focusing on miR-21 with data available. Our meta analysis on single miR-21 indicated that the pooled sensitivity was 0.90 (95%CI 0.82–0.94) and the pooled specificity was 0.72 (95%CI 0.57–0.83). The area under the SROC was 0.91 (95%CI 0.88–0.93; Figure 7). Those findings suggest that a single miR-21 can be a good indicator for pancreatic cancer diagnosis.

**Figure 8:**
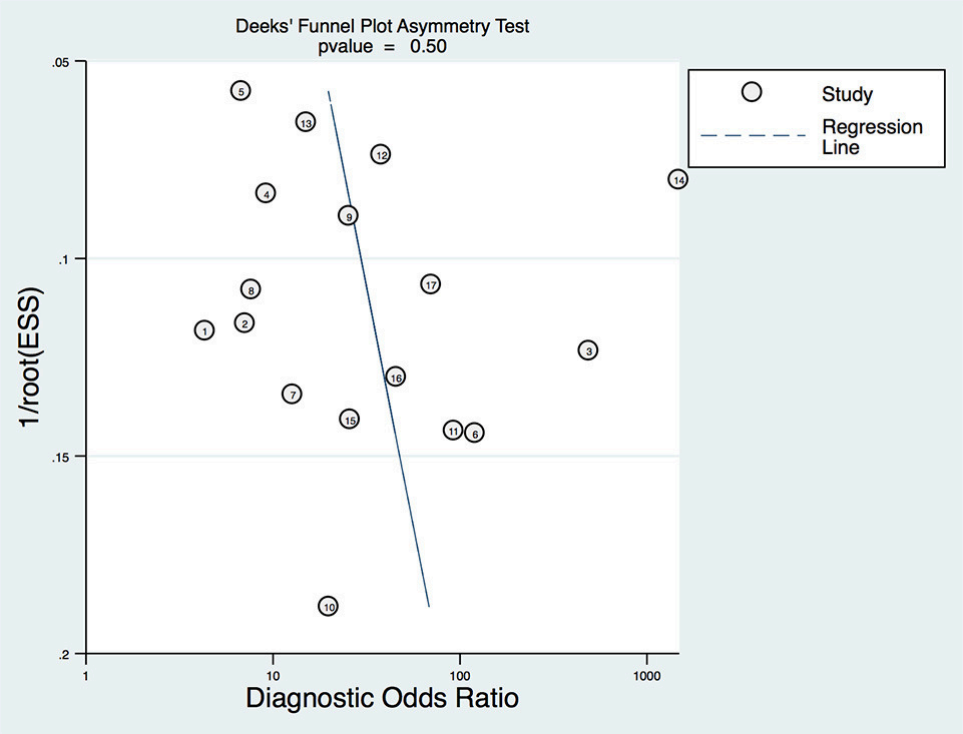
Deek's funnel plot for the assessment of publication bias.

To explore any potential publication bias of the studies included in this meta-analysis, the Deek's funnel plot with the asymmetry test was generated, and the *p* value was 0.7, suggesting no publication bias existed in the included studies (Figure 8).

## Discussion

At present, the diagnosis of malignant tumors depends mostly on medical imaging, but the associated sensitivity and specificity are poor, leading to tumors not being diagnosed in the early stage of cancer. As a result, most patients with malignant tumors are diagnosed in a later stage. With a continually increasing mortality rate, pancreatic cancer has become a health and economic burden for patients and their family members. Therefore, there is an urgent need to find more sensitive, non-invasive biomarkers to facilitate the diagnosis of pancreatic cancer (Goh and Yoon, 2012).

miRNAs are involved in the development and progression of various diseases, especially malignant tumors. Some studies have confirmed that miRNAs can be stable in bodily fluids for cancer detection. In the development of the pancreas and the onset of pancreatic neoplasm, miRNAs are important regulators and mediators. Adopting specific miRNAs as biomarkers for early diagnosis of pancreatic cancer has potential implications for clinicians and patients.

In this review, 17 eligible articles with a total of 1916 cases were identified through database and manual searches and included in a meta-analysis. According to QUADAS-2 quality evaluation, all the included studies were of middle-to-high quality. Meta-analysis showed that the combined sensitivity, specificity, and DOR for the miRNA profiles in the 17 studies were 88%, 83%, and 36.7, respectively. The PLR and NLR were 4.3 and 0.16, respectively. The PLR suggests that a patient with pancreatic cancer is 4.3 times more likely to have positive results on miRNA tests than a normal person and the NLR suggests that the probability of a false negative result basted on miRNA signature detection was 16%. Additionally, the AUC of the combined ROC curve for circulating miRNAs in diagnosing pancreatic cancer was 0.92, suggesting that the circulating miRNA profile is of intermediate efficacy for the diagnosis of pancreatic cancer. A subgroup analysis of sources of heterogeneity revealed that there were no significant differences the sample size and patient ethnicity among the included studies. Therefore, the results of this quantitive study indicate that the circulating miRNA signature has sufficient accuracy for the diagnosis of pancreatic cancer.

To further explore the value of a single miRNA in pancreatic cancer diagnosis, miR-21 was examined. The previous evidences also suggested that miR-21 and itsdownstream targets play a key role in cell proliferation, apoptosis and cell cycle and mediate oncogenic function in pancreatic cancer (Park et al., 2009; Bhatti et al., 2011). The results of meta analysis indicate that the diagnostic value of of miR-21 was relatively higher than a panel of miRNAs. According to these data, the circulating miR-21 may be a promising diagnostic biomarker for diagnosing pancreatic cancer.

There are some limitations in this study. First, the limited number of patients and controls may affect the results of meta-analysis. Some studies were excluded due to the lack of sufficient data collected during the screening process. Secondly, the purpose of this study was to investigate the effectiveness of the circulating miRNA signature as a biomarker of pancreatic cancer, but samples for other types of cancer may affect the interpretation of the results of the meta-analysis. Finally, in the included studies, the differences in the duration of sample storage, miRNA extraction methods, and miRNA detection methods could influence the results of the meta-analysis. The miRNA transcript levels were affected by the different RNA processing methods. Moreover, the studies utilized different methods for quantitative detection and normalization in miRNA analysis, which may account for the differences reported across different studies.

Adopting a proper miRNA signature for individual diagnosis in clinical practice is still a great challenge. Further studies are needed for validation, even though the results in the current analysis showed that the miRNA expression profile can be used as a diagnostic indicator of pancreatic cancer. Moreover, miRNAs provide new targets and indicators for developing cancer therapies and predicating prognosis of pancreatic cancer, which is of great significance to improving the survival of patients and increasing the expected life expectancy and quality of life of patients with pancreatic cancer.

In conclusion, the present meta-analysis confirms a potential role for miRNA detection in pancreatic cancer diagnosis. Moreover, further studies are required to confirm molecular mechanism of the dysregulated circulating miRNA signature in pancreatic cancer, and prospective, multi-centered, large-scale clinical trials will further aid the establishment of miRNA combinations as diagnostic biomarkers for pancreatic cancer and elucidate the pathophysiological interactions between miRNAs and pancreatic pathology (Firpo et al., 2014).

## Author contributions

XL and PG did the literature research, data acquisition, statistical analysis, and wrote the manuscript. YW provided insightful thoughts to study concepts and wrote the manuscript. XW designed the study, revised the manuscript, and approved the final version.

### Conflict of interest statement

The authors declare that the research was conducted in the absence of any commercial or financial relationships that could be construed as a potential conflict of interest.
